# Patent foramen ovale occlusion with the Cocoon PFO Occluder. The PROS-IT collaborative project

**DOI:** 10.3389/fcvm.2022.1064026

**Published:** 2023-01-11

**Authors:** Luca Testa, Antonio Popolo Rubbio, Mattia Squillace, Flavio Albano, Vincenzo Cesario, Matteo Casenghi, Giuseppe Tarantini, Paolo Pagnotta, Alfonso Ielasi, Grigore Popusoi, Leonardo Paloscia, Alessandro Durante, Diego Maffeo, Francesco Meucci, Giuliano Valentini, Gian Paolo Ussia, Paolo Cioffi, Bernardo Cortese, Giuseppe Sangiorgi, Gaetano Contegiacomo, Francesco Bedogni

**Affiliations:** ^1^Department of Cardiology, IRCCS Policlinico San Donato, Milan, Italy; ^2^Department of Cardiac, Thoracic and Vascular Sciences, University of Padua, Padua, Italy; ^3^Cardio Center, Humanitas Research Hospital, Milan, Italy; ^4^Clinical and Interventional Cardiology Unit, Istituto Clinico Sant'Ambrogio, Milan, Italy; ^5^Interventional Cardiology Service, Montevergine Clinic, Mercogliano, Italy; ^6^U.O.C. Cardiologia-UTIC, Ospedale Santo Spirito, Pescara, Italy; ^7^Cardiology Unit, Policlinico San Marco, Bergamo, Italy; ^8^Fondazione Poliambulanza, Brescia, Italy; ^9^Structural Interventional Cardiology, Careggi University Hospital, Florence, Italy; ^10^Cardiology Intensive Care Unit and Cath Lab, Ospedale Civile SS. Filippo e Nicola, L'Aquila, Italy; ^11^Unit of Cardiovascular Science, Department of Medicine, Campus Bio-Medico University, Rome, Italy; ^12^Department of Cardiology, Cardiac Cath Lab, Città di Alessandria Institute, Alessandria, Italy; ^13^Department of Cardiology, San Carlo Clinic, Milan, Italy; ^14^Department of Biomedicine and Prevention, Tor Vergata University of Rome, Rome, Italy; ^15^Department of Interventional Cardiology, Anthea Hospital, GVM Care and Research, Bari, Italy

**Keywords:** patent foramen ovale (PFO), embolism, translational, nanoparticle, platinum

## Abstract

**Background:**

The Cocoon patent foramen ovale (PFO) Occluder is a new generation nitinol alloy double-disk device coated with nanoplatinum, likely useful in patients with nickel hypersensitivity. Early results and mid-term outcomes of this device in percutaneous PFO closure are missing.

**Aims:**

To assess the preliminary efficacy and safety profile of PFO closure with Cocoon device in an Italian multi-center registry.

**Methods:**

This is a prospective registry of 189 consecutive adult patients treated with the Cocoon PFO Occluder at 15 Italian centers from May 2017 till May 2020. Patients were followed up for 2 years.

**Results:**

Closure of the PFO with Cocoon Occluder was carried out successfully in all patients, with complete closure without residual shunt in 94.7% of the patients and minimal shunt in 5.3%. Except from a case of paroxysmal supraventricular tachycardia and a major vascular bleeding, no procedural and in-hospital device-related complications occurred. No patient developed cardiac erosions, allergic reactions to nickel, or any other major complications during the follow-up. During the follow-up period, 2 cases of new-onset atrial fibrillation occurred within thirty-day.

**Conclusions:**

Percutaneous closure of PFO with Cocoon Occluder provided satisfactory procedural and mid-term clinical follow-up results in a real-world registry.

## Introduction

The presence of patent foramen ovale (PFO) can be detected in about 25% of the adult population, with implication in the pathogenesis of different medical conditions as cryptogenic stroke, decompression illness, platypnea-orthodeoxia syndrome and, although still controversial, migraine with aura (1–3). When PFO closure is indicated, percutaneous closure is recommended as the method of choice (4–6). In this regards, multiple observational studies, meta-analyses and trials have shown a benefit of percutaneous PFO closure, demonstrating favorable long-term results in terms of efficacy in preventing recurrence of stroke, improvement in quality of life and cost-effectiveness of this procedure when compared to medical therapy, thus increasing the popularity of this procedure (7–12).

Nowadays, different systems are available for percutaneous PFO closure, including a suture-based system (13, 14). Percutaneous PFO closure is considered a relatively simple procedure but, although very rarely, it has a potential risk of cardiac erosion, and nickel allergic reactions in predisposed subjects (15, 16).

The Cocoon PFO Occluder (Vascular Innovations Co. Nonthaburi, Thailand) was recently released as a novel nitinol alloy double-disk device, with a similar design to Amplatzer Occluder device (Abbott, Abbott Park, Illinois, USA). The Cocoon PFO Occluder features a specific nanoplatinum coating that should abolish the issue of nickel hypersensitivity (Ni-Hy) and smooth the microscopic geometry of the disks to minimize the risk of erosion. Initial evaluation of Cocoon septal Occluder for closure of atrial septal defects in adults and pediatric patients was recently presented, whereas procedural and follow-up data of the Cocoon PFO Occluder are missing (17).

We herein feature a nation-wide registry concerning the acute procedural data and the mid-term clinical follow-up of adult patients with PFO treated with the Cocoon Occluder.

## Methods

### Patient population and study design

In this multi-center, observational registry, adult patients ≥18 years old, with a clinical indication for PFO closure according to the current guidelines, treated with the Cocoon PFO Occluder were consecutively included (4–6). All the patients treated with Cocoon PFO Occluder at the participating centers were sequentially included in the registry in a prospective way and data where then analyzed retrospectively.

Patients were eligible for the procedure if they presented a documented history of cryptogenic stroke (radiologically verified) or transient ischemic attack (TIA) in the previous 12 months, intractable migraine or need for PFO closure for professional reasons. Exclusion criteria were subjects with previous stroke or TIA with known etiology or with congenital or pre-existing neurological disorders (i.e., multiple sclerosis, epilepsy) or intra-cranial disease; subjects with atrial fibrillation/flutter or other known emboligenic heart diseases; subjects with carotid, vertebral or basilar artery stenosis >50%; subjects with previous endocarditis or at risk for endocarditis; subjects with contraindications to aspirin, clopidogrel or anticoagulant therapy; subjects under tutorship, curatorship or unable to take the prescribed medical therapy.

Baseline and procedural variables were collected from medical records through a web-based electronic case report form from each participating center. Follow-up data were then obtained by means of telephone calls and/or outpatients clinical visits.

For the purpose of our analysis, we considered as efficacy outcome the occurrence of effective PFO closure with a residual minimal RLS or lower in acute and at the follow-up. As regards safety outcome, we considered the occurrence of peri-procedural major adverse events (MAEs) including death, acute neurological disorders, new-onset arrhythmia, major bleeding, cardiac tamponade or complications related to the vascular access, device malposition and embolization. The secondary safety outcome was a composite of MAEs detected during the follow-up including arrhythmia, late neurological disorders, endocarditis and device-related adverse events (malposition, embolization, thrombosis, and systemic nickel allergy syndrome (SNAS).

Before the procedure, all the patients underwent contrast-enhanced transcranial Doppler ultrasound or transesophageal echocardiography with bubble test, in order to confirm the presence of the PFO and the severity of RLS. The grading of RLS through the PFO was semi-quantitative in accordance to the number of micro-bubbles detected in left atrium at rest and after Valsalva maneuver and defined as severe (≥20 bubbles or opacification), moderate (6–19 bubbles), minimal (1–5 bubbles) or no-shunt (0 bubbles) (18).

All the patients signed an informed written consent.

The registry was approved by the local ethical committees of each participating hospital and the study complied with the Declaration of Helsinki.

### Device

**Figure 1:**
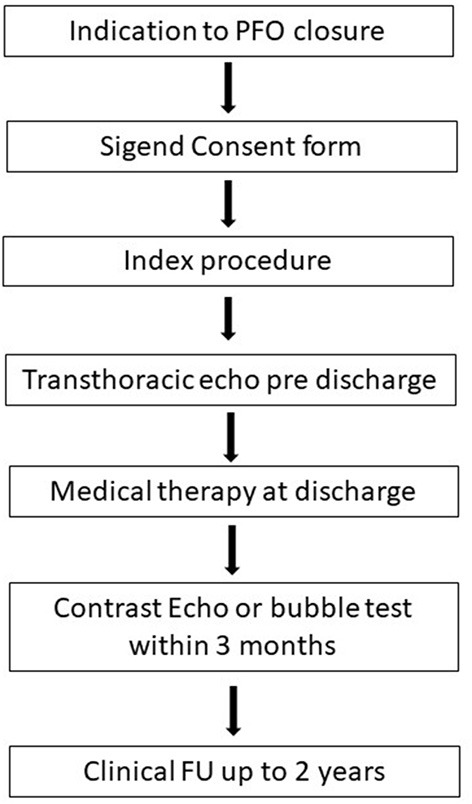
Study design flow chart.

The Cocoon PFO Occluder is an implantable self-expandable double-disk device, structurally similar to the Amplatzer Occluder device (13, 19). The two disks are linked together by a short connecting waist allowing free motion of each disc. The disc diameter range is 18 to 30 mm for the left atrium disc and 18 to 35 mm for the right atrium disc, set up in different combinations (Figure 1). Similarly to other devices for PFO closure, the Cocoon PFO Occluder presents a nitinol core structure, but the main difference lies in the nanoplatinum coating of its wire mesh, with platinum atoms up to 25 microns using nanofusion technology. According to its manufacturer's instructions for use, the nanoplatinum coating may prevent nickel leaching into the bloodstream and increase device-surface smoothing.

### Procedure

**Figure 2:**
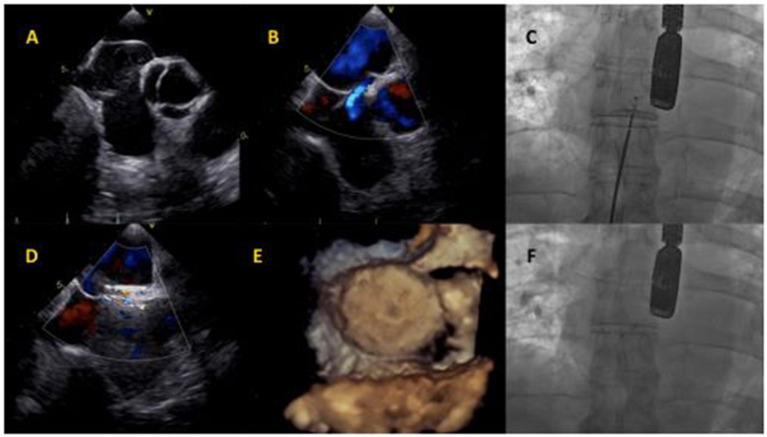
Cocoon PFO Occluder implantation. **(A)** Baseline transesophageal echocardiography showing atrial septum aneurysm and Chiari network. **(B)** Baseline interatrial shunt. **(C)** Cocoon PFO Occluder positioning at fluoroscopy. **(D)** Intraprocedural transesophageal echocardiography, showing minimal intra-device shunt. **(E)** Device assessment with 3D-echocardiography imaging. **(F)** Final device release at fluoroscopy.

All patients underwent the procedure under general anesthesia or conscious sedation, and were simultaneously studied by transesophageal echocardiography or intra-cardiac echocardiography, at discretion of each participating center. The procedure was performed as for standard for percutaneous PFO closure, with wire crossing through the PFO into the left upper pulmonary vein and delivery sheath advancing over the stiff wire, with final release of the intended device; an illustrative case is displayed in Figure 2. The patients were heparinized to achieve an activated clotting time of more than 250 s during the whole procedure. The size of the device has been selected according to the echocardiographic septum and tunnel characteristics or according to the balloon sizing and rim adequacy. When device position was optimal, the device was released by a counterclockwise rotation of the delivery cable. Also in this case, residual RLS were graded as severe (≥20 bubbles or opacification), moderate (6–19 bubbles), minimal (1–5 bubbles) or no-shunt (0 bubbles).

The patients were discharged with aspirin and/or clopidogrel and/or oral anticoagulant in accordance to their clinical history and at discretion of the physicians. In order to evaluate residual RLS, contrast-enhanced transthoracic echocardiography and transcranial Doppler follow-up were done within 3 months post procedure.

Clinical FU was evaluated and recorded up to 2 years.

### Statistical analysis

Categorical and dichotomous variables were expressed as absolute numbers and percentages whereas continuous variables were expressed as mean±standard deviation (SD) or median (25^th^ to 75^th^ interquartile range–IQR), as appropriate according to their distribution. Continuous variables were analyzed for normal distribution using the Shapiro-Wilk test. The data were analyzed with SPSS statistics software (version 25, IBM Corp., Armonk, N.Y., USA).

## Results

### Baseline characteristics

Between May 2018 and May 2020, at 15 Italian centers, 189 patients with clinical indications for percutaneous PFO closure were treated with Cocoon PFO Occluder. Complete patient characteristics are listed in Table 1.

**Table 1:** Baseline demographic and clinical characteristics of the study population.

	***N* = 189**
**Baseline characteristics**	
Age, years	50 ± 13
Female, *n* (%)	96 (50.8)
BMI, Kg/m^2^	24 (22–27)
Hypertension, *n* (%)	49 (25.9)
Diabetes, *n* (%)	4 (2.1)
Smoker, *n* (%)	18 (9.5)
Coagulation Disorders, *n* (%) • Hyperhomocysteinemia, *n* (%) • Protein C/S Deficiency, *n* (%) • Leiden V Factor Mutation, *n* (%) • Factor II Mutation, *n* (%) • MTHFR Mutation, *n* (%) • Antiphospholipid Syndrome, *n* (%) • Others, *n* (%)	14 (7.4) 5 (2.6) 2 (1.1) 1 (0.5) 1 (0.5) 1 (0.5) 1 (0.5) 3 (1.6)
Atrial septal aneurysm, *n* (%)	91 (48.1)
Family history for congenital heart disease, *n* (%)	5 (2.6)
Congenital heart disease, *n* (%)	2 (1.1)
Rope score 1–3	5%
Rope score 4–6	15%
Rope score >6	80%
**Shunt at rest**	
None (0 bubbles), *n* (%) Minimal (1–5 bubbles), *n* (%) Moderate (6–19 bubbles), *n* (%) Severe (≥20 bubbles), *n* (%)	40 (21.2) 49 (26.0) 35 (18.5) 65 (34.4)
**History and clinical presentation**	
Cryptogenic stroke, *n* (%)	80 (42.3)
Multiple cryptogenic stroke, *n* (%)	7 (3.7)
TIA, *n* (%)	61 (32.3)
Multiple TIA, *n* (%)	5 (2.6)
Paradoxical embolism, *n* (%)	7 (3.7)
Deep vein thrombosis, *n* (%)	3 (1.6)
Migraine, *n* (%)	52 (27.5)
Professional Reasons, *n* (%)	3 (1.6)

Categorical variables are expressed as n (%). Continuous variables are expressed as mean ± standard deviation (SD) or median and interquartile range (IQR), as appropriate.

BMI, Body Mass Index; TIA, Transient Ischemic Attack.

The mean age was 5 ± 13 years and there was a slight prevalence of female gender (50.8%). Coagulation disorders were present in just 7.4% of patients. The main indications for PFO closure were cryptogenic stroke (42.3%) and TIA (32.3%). The basal shunt was severe in 65 patients (34.4%) and almost half of the patients presented atrial septum aneurysm (48.1%).

### Procedural characteristics

The procedure was performed in conscious sedation in 67.2% of patients (Table 2). Transesophageal echocardiography was the main imaging modality (80.4%). The size of the device was selected according to the echocardiographic septum characteristics (88.9%) or according to the balloon sizing (11.1%). Mean procedural time was 34 ± 20 min. The most used device was Cocoon PFO Occluder 25^*^18 mm (73.0%). During the procedure, except a single case of paroxysmal supraventricular tachycardia and a major vascular bleeding (patient was on clopidogrel), no other significant adverse events occurred. Device embolization and recapturing or procedural cardiac erosions neither occurred. Post-procedural residual RLS was absent in most of the patients (94.7%), whereas a minimal RLS was documented in 5.3%.

**Table 2:** Procedural characteristics.

	***N* = 189**
**Procedural variables**	
Conscious sedation, *n* (%)	127 (67.2)
General anesthesia, *n* (%)	62 (32.8)
Procedural time, min	34 ± 20
Transesophageal echocardiography, *n* (%)	152 (80.4)
Intracardiac Echocardiography, *n* (%) Balloon sizing, *n* (%)	37 (19.6) 21 (11.1)
Intra-Procedural MAEs • Death • Stroke/TIA • Atrial fibrillation • Paroxysmal supraventricular tachycardia • Ventricular tachycardia/fibrillation • Major bleeding • Cardiac tamponade • Device malposition • Device embolization	0 (0.0) 0 (0.0) 0 (0.0) 1 (0.5) 0 (0.0) 1 (0.5) 0 (0.0) 0 (0.0) 0 (0.0)
**Device employed**	
• Cocoon 18*18 mm, *n* (%)	12 (6.3)
• Cocoon 25*18 mm, *n* (%)	138 (73.0)
• Cocoon 25*25 mm, *n* (%)	4 (2.1)
• Cocoon 30*30 mm, *n* (%)	28 (14.8)
• Cocoon 35*25 mm, *n* (%)	7 (3.7)
**Residual shunt**	
• None (0 bubbles), *n* (%)	179 (94.7)
• Minimal (1–5 bubbles), *n* (%)	10 (5.3)
• Moderate (6–19 bubbles), *n* (%)	0 (0.0)
• Severe (≥20 bubbles), *n* (%)	0 (0.0)

Categorical variables are expressed as n (%). Continuous variables are expressed as mean ± standard deviation (SD) or median and interquartile range (IQR), as appropriate.

MAEs, Major Adverse Events; TIA, Transient Ischemic Attack.

### Post-procedural outcome

No adverse events occurred during the hospital stay. Most of the patients were discharged with indication to dual antiplatelet therapy with aspirin and clopidogrel (97.9%), for 1–12 months (Table 3). Four patients received a short- single antiplatelet therapy with aspirin and 5 patients received an oral anticoagulant therapy in association with antiplatelet: the history of a coagulation disorder was the reason to combine the anticoagulant with the antiplatelet therapy. This therapy was kept for a month and then followed by the anticoagulant therapy alone.

**Table 3:** Discharge medical therapy.

	***N* = 189**
**Medical therapy**	
Aspirin and clopidogrel • 1 Month • 3 Months • >3 Months Single aspirin Anticoagulant therapy	185 (97.9) 54 (28.5) 120 (64.8) 11 (6.7) 4 (2.1) 5 (2.6)
• Vitamin K antagonist • Apixaban • Dabigatran • Edoxaban • Rivaroxaban	1 (0.5) 1 (0.5) 0 (0.0) 2 (1.1) 1 (0.5)
Lifelong aspirin Lifelong clopidogrel Lifelong anticoagulant	51 (27%) 4 (2.1) 2 (1.1)

Categorical variables are expressed as n (%).

Life-long aspirin was prescribed in 27.0% of the cases, whereas life-long clopidogrel in 2.1%. Patients that were in treatment with anticoagulants before the procedure continued their standard treatment with vitamin K antagonists or direct oral anticoagulants.

The choice of the antiplatelet/anticoagulant regimen was left to the attending physician in order to mimic the real world, in the absence of clear-cut evidence on the most appropriate therapy after PFO closure.

Within 2 years post procedure, 2 cases (1.1%) of new-onset paroxysmal atrial fibrillation occurred, both within 3 weeks from the index procedure. No other major adverse events were reported. In all cases, contrast-enhanced echocardiographic and/or transcranial Doppler follow-up within 3 months after the index procedure showed absence of any severe RLS in all patients.

## Discussion

Percutaneous PFO closure is a well-established therapy for paradoxical left thromboembolism (5, 20). Multiple trials and meta-analyses showed the benefit of the double-disk technology PFO closure devices (13, 14). In this context, the Cocoon PFO Occluder has been introduced with the aim of surpassing the limitations of available devices but scarce data are available and the benefit of its technical features needs to be confirmed.

The main findings of this prospective nation-wide registry can be summarized as follows:

- The novel Cocoon PFO Occluder showed good results in terms of efficacy (with absence of severe residual RLS in 100% of the cases) and safety.
- The effective closure of the PFO was confirmed in all cases by means of appropriate imaging.
- No cardiac erosion, allergic reactions or thrombotic events occurred both in the early phase and during the 2 year FU.
- 2 cases (1.1%) of early new-onset paroxysmal atrial fibrillation occurred.

### Efficacy profile

The effectiveness of the Cocoon PFO Occluder in permanently closing the PFO seems to be consistent with those observed with the large RCTs concerning the Amplatzer Occluder and Gore Occluder (W. L. Gore and Associates, Inc, Newark, Delaware, USA) (6–9). Indeed, the Cocoon PFO Occluder obtained a complete resolution of the RLS in 94.7% of the cases and no ischemic events occurred up to 2 years post procedure. Previous literature concerning a limited number of cases consistently reported no recurrent events and no residual shunt over a 6-months follow-up (19, 20).

### Safety profile

The nanoplatinum coating of the Cocoon PFO Occluder is supposed to soften the structure of the device, and thus minimize the risk of cardiac erosion, that is a rare but dramatic complication, more often observed with devices for the treatment of ASD (16, 21). Whether the technical features of the device actually translate in a safer tool has to be demonstrated in a head to head comparison, however, we hereby report no cases of cardiac erosion or cardiac tamponade, which is in line with previous reports with this device (17).

#### Potential risk reduction of Ni-Hy

The specific design of Cocoon device may supposedly address the demands for device PFO closure in patients with Ni-Hy: the nanoplatinum coating theoretically prevent nickel ions release into the bloodstream. The Ni-Hy represents the classic presentation of a T cell-mediated, delayed-type hypersensitivity response to exogenous agents. The initial step is hapten binding to a skin carrier protein. The complex ultimately produces the sensitization of T cells. Sensitized T cells encountering the antigen at any time later will then lead to the release of cytokines, which in turn leads to macrophage activation and produces the immune response (22).

In literature, the incidence and real magnitude of the device-related Ni-Hy “syndrome” is still a matter of debate, and skin reactions must be distinguished from the systemic reactions which, in extreme situations, may lead to surgical explant: this critical situation always occur weeks to months after the device implant. Preliminary studies have shown that, during the first period of endothelization after Cocoon device implantation, there was no nickel release into the bloodstream (22–26).

A way to evaluate the presence of Ni-Hy might be the use of patches before the PFO closure, although it still unclear their role on top of clinical and physical examination (27).

Contact allergy secondary to the Amplatzer (St. Jude Medical, Inc., St. Paul, Minnesota), PFO-Star (Cardia Inc., Burnsville, Minnesota), and Gore Helex devices have been described previously (28–32).

In the 3 pivotal randomized trials on https://www.sciencedirect.com/topics/medicine-and-dentistry/patent-foramen-ovale PFO closure published in 2017, which included more than 2,000 patients, only 1 device-related allergic reaction was reported among the adverse events (7–9). No device explant was reported in any of the trials. Of note, patch testing to nickel was not required in the studies and patient reported history of nickel allergy was not an exclusion criterion for any of the trials.

In a retrospective analysis of explanation rates for PFO/ASD occluder devices, 38 of 13,736 (0.28%) of patients undergoing percutaneous closure had device removal (25). Allergy was not listed as the primary cause of explanation in any cases, but among the 14 patients who required device explanation for chest pain, 7 were found to have a positive patch test for nickel.

For people who present Ni-Hy, the use of suture-mediated system may be proposed, but this technology has an 18–20% risk of significant residual RLS (33, 34), thus its use has a weak rationale.

On the other hand, whether the use of a Cocoon is the appropriate solution has to be confirmed beyond the theoretical basis.

#### Potential risk reduction of device thrombosis

The polypropylene filling of the Coccon occluder may be associated with a reduced device-related thrombotic risk that, according to the literature, has been estimated with other devices between 1 and 2% (6, 17, 19).

Of note, despite 7.4% of patients in our registry presented coagulation disorders, no device-related thrombotic events were recorded in the peri-procedural setting, and up to 2 years.

In a recent publication, thrombus formation on the device was the justification for surgical excision in 4 (0.03%) of the 13,736 device implants. Of the 4 devices explanted with thrombus: 3 were CardioSEAL devices (0.15% of such devices) and 1 was an Amplatzer device (0.01%) (25).

A clear understanding of the risk of thrombosis related to different PFO closure devices is quite challenging as only a large scale direct comparison with other devices, with a comparable thrombotic risk and medical therapy might be reliable.

#### Arrhythmic complications

Major arrhythmic events have been previously reported in patients treated with occluder devices, with variable rates of 0.5–15%: apparently, there is a direct correlation with the size of the defect and, subsequently, with the size of the device selected that may cause a mechanical irritation and inflammatory reaction (6, 35, 36). In our registry, 1 case (0.5%) of paroxysmal supraventricular tachycardia occurred in the peri-procedural phase, whereas paroxysmal atrial fibrillation occurred after discharge within thirty-day in 2 patients (1.1%), both treated with a Cocoon PFO Occluder 25^*^18 mm. Due to the paroxysmal course of the arrhythmia, both cases were treated with antiarrhythmic drugs and life-long anticoagulant therapy. The incidence of AF after procedure seems to be lower than previous reports, likewise the incidence of supraventricular arrhythmias (37): overall, the lack of systematic ECG monitoring after the procedure may have led to an underestimation of the true incidence of arrhythmias.

### Post-implantation antiplatelet therapy

The type and duration of antiplatelet therapy was left to physician discretion to mirror the real world setting: most patients (97.9%) received dual antiplatelet therapy for a period varying from 1 to 12 months, although the vast majority of them were on dual antiplatelet therapy for 1 to 3 months, and only 4 patients received a short-term single antiplatelet therapy, whereas 5 patients received anticoagulant therapy in association. This variability confirms the lack of a consensus about the pharmacological post-implantation approach, and highlights the need for more evidences (38–40).

## Limitations

This is a prospective single arm registry and the endpoints were reported by the participating centers. The sample size of this study is relatively small, but, to the best of our knowledge, this is the largest experience so far concerning the real-world preliminary efficacy and safety profile of this new technology. The latter seems at least comparable with available and more extensively evaluated devices; the theoretical benefit in terms of increased safety has to be demonstrated.

## Conclusion

The Cocoon Occluder device showed a good performance in terms of shunt resolution and recurrence of neurological ischemic events. The low rate of peri-procedural complications supports the use of the Cocoon PFO Occluder in patients with indication to PFO closure. Larger scale and comparative studies with long-term follow-up are needed in order to confirm whether the technical features of this new technology actually translate into an improved safety/efficacy profile.

## Data availability statement

The raw data supporting the conclusions of this article will be made available by the authors, without undue reservation.

## Ethics statement

The studies involving human participants were reviewed and approved by each participating center EC. The patients/participants provided their written informed consent to participate in this study.

## Author contributions

LT and AP drafted and revised the manuscript. AP conducted the statistical analysis. LT and FB made substantial expert contributions. All authors contributed to the data collection. All authors contributed to the article and approved the submitted version.
